# Hematopoietic and lymphatic cancers in patients with periodontitis: a systematic review and meta-analysis

**DOI:** 10.4317/medoral.23166

**Published:** 2019-12-24

**Authors:** Yougen Wu, Xiaojun Shi, Yinghua Li, Ju Xia, Yuting Gu, Qingqing Qian, Yang Hong

**Affiliations:** 1 National Institute of Clinical Research, The Fifth People's Hospital of Shanghai, Fudan University, Shanghai 200240, China; 2 Department of Stomatology, The Fifth People's Hospital of Shanghai, Fudan University, Shanghai, 200240, China; 3 Central Laboratory, The Fifth People's Hospital of Shanghai, Fudan University, Shanghai 200240, China; 4 Department of Pharmacy, The Fifth People’s Hospital of Shanghai, Fudan University, Shanghai 200240, China; 5 Department of Osteology, The Fifth People’s Hospital of Shanghai, Fudan University, Shanghai 200240, China

## Abstract

**Background:**

Numerous studies have explored the correlation of periodontal disease (PD) with risk of hematopoietic and lymphatic cancers, but the findings were inconsistent. Therefore, we did a meta-analysis to ascertain the correlation of PD with risk of incident hematopoietic and lymphatic cancers.

**Material and Methods:**

The authors searched relevant studies in databases (PubMed, Web of Science, and MEDLINE). The summary relative risk (RR) along with 95% confidence interval (CI) was calculated by use of random or fixed effects models.

**Results:**

Six studies were included in qualitative synthesis. The pooled analysis revealed that PD was significantly associated with an increased risk of hematopoietic and lymphatic cancers (RR = 1.17; 95% CI = 1.07–1.27; *P* = 0). Stratified analysis showed the association of PD with hematopoietic and lymphatic cancers remained significant in the never smokers (RR = 1.28; 95% CI = 1.07–1.54; *P* = 0.007), and in the American population (RR = 1.17; 95% CI = 1.05–1.30; *P* = 0.003), respectively.

**Conclusions:**

Never smokers population and the American population with PD have a higher risk of developing hematopoietic and lymphatic cancers. PD might be considered as a risk factor for hematopoietic and lymphatic cancers.

** Key words:**Periodontal disease, hematopoietic and lymphatic cancer, meta-analysis, systematic review.

## Introduction

PD is a chronic inflammatory condition that affects the tissues surrounding the teeth, including alveolar bone, cementum, periodontal ligament and gingival. If periodontitis remains untreated, it may lead to annihilation of periodontium, tooth looseness and ultimately tooth loss. PD has been reported to increase the risk of chronic diseases, such as diabetes, cardiovascular disease, rheumatoid arthritis, and pulmonary disease (1-5). Furthermore, increasing evidence indicates that people with periodontitis may be at higher risk of developing oral cancers (6), cancer of head and neck (7-8), esophagus (9), lung (4), pancreas (10), colorectum (11), and breast (12). Additionally, greater number of tooth loss might increase the risk of developing cancer in patients.

Hematopoietic and lymphatic malignancies are cancers that affect blood, lymphatic system, and bone marrow. There are a number of risk factors associated with lymphoma (Hodgkin lymphoma, non-Hodgkin lymphoma), such as age, gender, Epstein-Barr infection, and immune dysregulation (https://www.cancer.gov/types/lymphoma). The most common risk factors associated with hematopoietic cancers (acute lymphoblastic leukemia, acute myelogenous leukemia, chronic lymphocytic leukemia, chronic myelogenous leukemia, multiple myeloma) include older age, smoking, prior chemotherapy, and exposure to radiation (https://www.cancer.gov/types/leukemia).

Recently, numerous epidemiological studies exploring the risk of hematopoietic and lymphatic cancers in patients with periodontitis have been published (13-19). However, these studies showed inconsistent results and the evidence remained inclusive. Therefore, we aimed to systematically evaluate the published literature and to quantify the association of periodontitis with risk of hematopoietic and lymphatic cancers via meta-analysis.

## Material and Methods

- Literature and search strategy

We followed the instructions of the PRISMA to conduct this meta-analysis (20). The focused question followed the PECO (population, exposure, comparison, outcomes) criteria: The population (P) was patients of any age; the exposure (E) was the presence of periodontal diseases (PDs); the comparison (C) was the absence of PDs; and the outcomes (O) were patients diagnosed with hematopoietic or lymphatic cancers.

PubMed, Web of Science, and MEDLINE were systematically searched to identify potentially eligible studies that assessed the correlation of PDs with hematopoietic and lymphatic cancers. We used the search terms: (“periodontal diseases” or periodontitis or gingivitis or “tooth loss” or “teeth loss”) and (hematological or haematological or hematologic or hematopoietic or lymphatic or lymphomas or lymphoma or lymphoid) and (cancers or malignancies). The search was completed on 31 Oct 2018.

- Selection criteria, data extraction and quality assessment

Published studies that estimated the relative risk of hematopoietic and lymphatic cancers associated with any measure of PD (i.e. periodontitis, gingivitis) were eligible. Comments, review articles and meta-analysis were excluded.

The following data from each included study were independently extracted by two investigators (YGW and XJS). The relevant data included first author of the study, year of publication, country of origin, study design, sample sizes in each group, age, gender, cancer type, duration of follow-up, exposure, relative risk (RR), hazards ratios (HR), or odds ratio (OR) estimates with 95%CI for outcomes.

The quality of included studies was assessed according to the Newcastle-Ottawa Scale (NOS) (21). This scale consists of assessment of three parts: selection (0-4 points), comparability (0-2 points), and outcome/exposure (0-3 points) for a total score of 9 points. In the NOS, poor, moderate, and high quality was scored 0–3, 4–6, and 7–9, respectively.

- Statistical analysis

Relative risk (RR) with 95% confidence interval (CI) was used as the common measure of association across studies. RR was calculated by the formula RR = (event/total)exposure/(event/total)non-exposure, in which (event/total)exposure and (event/total)non-exposure is the incidence of an outcome of interest in the exposed group and non-exposed group, respectively. HR was considered equivalent to RR when pooled in qualitative synthesis. When the incidence of outcome is more than 10% in a cohort study, the OR was transformed into RR with this formula: RR = OR/[(1 – P0) + (P0 × OR)], in which P0 is the incidence of the outcome of interest in the non-exposed group (22). The standard error (SE) of the converted RR was determined with this formula: SElog(RR) = SElog(OR) × log(RR)/log(OR) (23).

Heterogeneity across studies was evaluated using the Q statistic (*P* < 0.10 was regarded as statistical significance) and was quantified with I2 statistic. I2 values higher than 50% indicated moderate heterogeneity. In the presence of heterogeneity (*P* < 0.10), we combined the effect estimates across studies by use of a random-effects model (24); otherwise, the fixed effect model was adopted (25). The potential heterogeneity across studies was explored by subgroup analysis and sensitivity analysis. Sensitivity analysis was conducted via deleting each study at a time and calculating the summary effect size of the remaining studies. The funnel plots as well as Egger regression test were applied to assess publication bias (26). We used STATA 14.0 (Stata Corporation, College Station, TX, U.S.A.) for data analysis. Two-sided *P* < 0.05 was regarded as statistically significant.

## Results

- Literature retrieved and study characteristics

**Figure 1:**
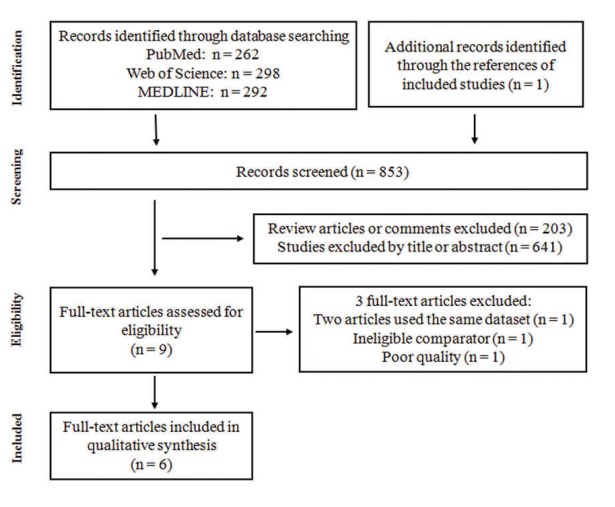
Flow diagram for study selection in this meta-analysis.

A total of 853 articles were initially identified and assessed. Fig. 1 showed the detailed ﬂow diagram of the exclusion and inclusion process. Two articles used the same dataset so the more comprehensive analysis by Bertrand *et al* (16) was included and the study by Michaud *et al* (27) was excluded from the quantitative synthesis. The study by Dizdar *et al* was excluded for poor quality. The study by WEN *et al* (28) was also excluded because of ineligible comparator. Six articles ultimately were eligible for inclusion and were included in qualitative synthesis (13-18). Five of the six studies were cohort studies (14-18), and one study (13) was case-control study.

Table 1 summarized the detailed characteristics of included studies reporting risk estimates for the correlation of PD with hematopoietic and lymphatic cancers. Quality assessment of the included studies according to the NOS is shown in Table 2 and Table 3. All of the six studies were of high quality scoring ≥ 7.

**Table 1:**
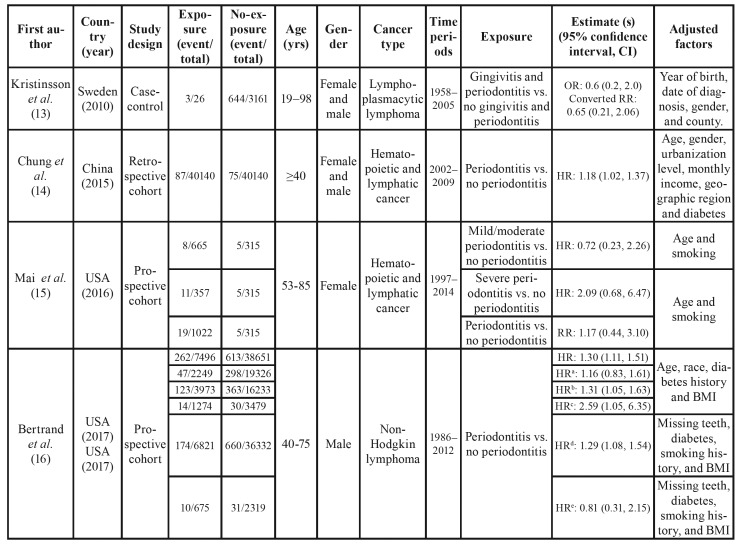
Characteristics of studies on the association of periodontal disease with hematopoietic and lymphatic cancers.

**Table 1 cont.**
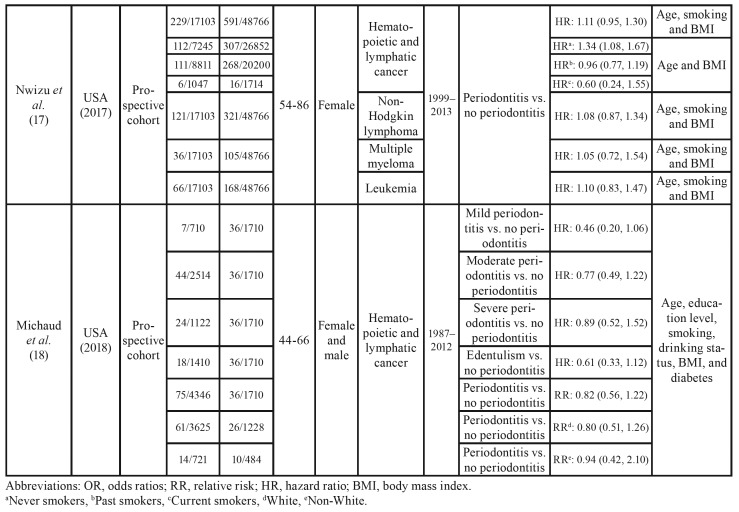
Characteristics of studies on the association of periodontal disease with hematopoietic and lymphatic cancers.

**Table 2:**
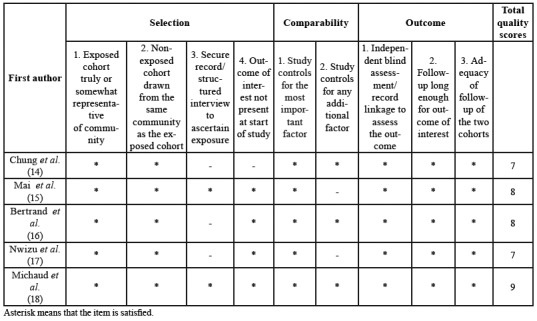
Quality assessment of cohort studies according to the Newcastle-Ottawa scale.

**Table 3:**
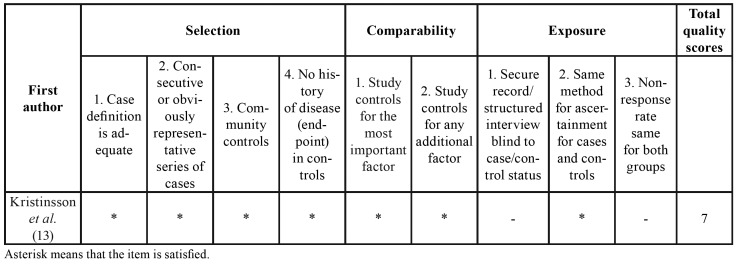
Quality assessment of case-control studies according to the Newcastle-Ottawa scale.

- Overall estimates and subgroup analysis

**Figure 2:**
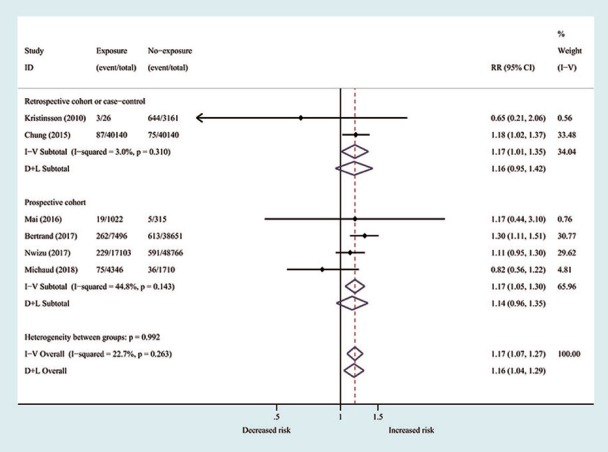
Forest plot for periodontal disease associated with hematopoietic and lymphatic cancers risk. The relative risk with 95% confidence interval for each study is represented by the square and horizontal line, respectively. The diamonds show the estimated pooled relative risk with corresponding 95% confidence interval.

Pooled results showed that subjects with PD have an increased risk for hematopoietic and lymphatic cancers (RR = 1.17; 95 % CI 1.07–1.27; *P* = 0). No evidence of heterogeneity was observed (*P* = 0.263; I2 = 22.7%; Fig. 2). Table 4 showed the results of subgroup analysis by study type, study design, smoking status, gender, country origin, ethnicity, and stage of PD. Results of the subgroup analyses based on cancer types indicated that patients with PD increased the risk of lymphatic cancers (RR = 1.21; 95% CI = 1.07–1.37), while the risk of hematopoietic cancers was not significant (RR = 1.08; 95% CI = 0.86–1.36). Subgroup analyses according to study design revealed significant relation of PD with increased risk of hematopoietic and lymphatic cancers in prospective cohort studies (RR = 1.17; 95% CI = 1.05–1.30) and in retrospective studies (RR = 1.17; 95% CI = 1.01–1.35).

**Table 4:**
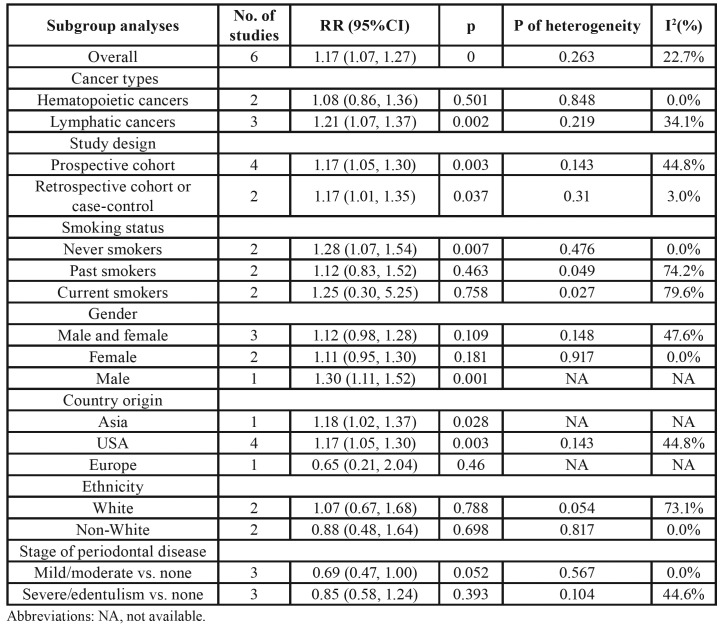
Results of subgroup analyses of pooled relative risk (RR) and 95% confidence interval (CI).

Analyses stratified by smoking status showed that PD was associated with risk of hematopoietic and lymphatic cancers among never smokers (RR = 1.28; 95% CI = 1.07–1.54) but not among past (RR = 1.12; 95% CI = 0.83–1.52) or current smokers (RR = 1.25; 95% CI = 0.30–5.25). A gender-specific analysis showed that the association between PD and risk of hematopoietic and lymphatic cancers was not statistically significant in male and female population (summary RR = 1.12; 95% CI = 0.98–1.28), and female population (pooled RR = 1.11; 95% CI = 0.95–1.30), respectively. Four studies were performed in America, one in Asia, and one in Europe. For subgroup analysis by geographic region, the significant association between PD and risk of hematopoietic and lymphatic cancers was observed among the American population (RR = 1.17; 95% CI = 1.05–1.30). Stratification analysis by ethnicity demonstrated no significant association between PD and risk of hematopoietic and lymphatic cancers in the white population or non-white population. Moreover, pooled estimates found no evidence of increased risk of hematopoietic and lymphatic cancers among individuals with mild/moderate PD (RR = 0.69; 95% CI = 0.47–1.00) and severe/edentulism PD (RR = 0.85; 95% CI = 0.58–1.24), respectively.

- Sensitivity analyses

In sensitivity analysis using the leave-one-out approach, the results showed that no individual study significantly altered the pooled RR for PD on hematopoietic and lymphatic cancers risk, with pooled RR varying from 1.12 (95% CI, 1.01–1.24) to 1.20 (95% CI, 1.08–1.32), demonstrating the meta-analysis results were robust (Figure not shown).

- Bias assessment

**Figure 3:**
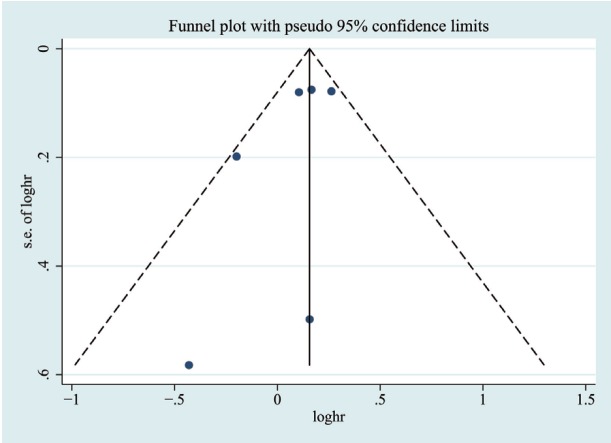
Funnel plot to assess publication bias. Each point indicates individual studies included in the meta-analysis.

To examine the extent of publication bias for included studies, funnel plot and Egger regression analysis were conducted. The funnel plot on included studies showed a little asymmetry (Fig. 3). The Egger regression analysis was not significant for publication bias across studies (*P* = 0.224).

## Discussion

The results on the association between PD and the risk of hematopoietic and lymphatic cancer are less consistent. No associations were detected in four of the six included studies (13,15,17,18), but two studies reported a higher risk of hematopoietic and lymphatic cancer among participants with PD (14,16). The qualitative synthesis results indicated that PD was significantly associated with increased risk of hematopoietic and lymphatic cancers, especially lymphatic cancers, which is in agreement with previous report (19).

The underlying mechanisms behind the potential association are as follows: first, PD may increase cancer risk through the chronic release of inflammatory mediators or immune system dysregulation (29-32). Second, PD may influence carcinogenesis via the increased exposure to carcinogenic nitrosamines (33). PD and poor dental hygiene were shown to enhance the formation of nitrosamines and oral bacteria in the oral cavity (34). Thus, anti-inflammation treatments for PD reduce markers of systemic inflammation and may decrease subsequent cancer risks. However, Hwang *et al*. found that anti-inflammation therapies of PD did not reduce the risk of hematopoietic and lymphatic cancers (35).

Tooth loss is often the result of severe periodontitis. Moreover, tooth loss was found to be positively associated with risk of certain cancers such as head and neck, esophageal, and lung cancers (34). A dose-response meta-analysis revealed that each ten-tooth loss was associated with a 3% increment of risk of hematopoietic cancer (36).

Our meta-analysis has several potential limitations. First, although most included studies have adjusted for common covariates, other unmeasured confounding factors such as socioeconomic status, stress, along with genetics might affect the correlation between PD and risk of hematological cancers (37). Second, we noted that the sample size was small and the results had a wide confidence interval in two studies (13,15), which limited the power of analyses. Moderate heterogeneity was detected among some subgroups. These analyses were not sufficiently powered owing to the limited number of studies included in the stratified groups. Thus, the subgroup results should be interpreted with caution. Finally, subgroup analysis revealed increasing severity of periodontitis was not associated with risk of hematopoietic and lymphatic cancers. Assessment methods of PD were heterogeneous in each study or the definitions for categories of PD severity varied between the included studies. Michaud *et al*., 2018 reported assessment of PD based on both level of attachment loss and pocket depth measurements (18), Mai *et al*., 2016 assessed PD severity using clinically measured alveolar crestal height (15), two studies used self-reported questionnaire for evaluating PD status (16,17), and two studies did not mention the procedures for assessment of PD (13,14), which might affect the heterogeneity and precision of our results.

The strengths of our pooled analysis are as follows. First, the meta-analysis is the first to exclusively explore the correlation of PD with risk of hematopoietic and lymphatic cancers. Second, we found low heterogeneity across studies for overall estimate, there was no publication bias. Subgroup and sensitivity analyses revealed consistent results for the relation of PD with risk of hematopoietic and lymphatic cancers, which imply the robustness and reliability of our findings. Third, most studies included in the meta-analysis were of high quality according to the predefined quality assessment criteria and controlled the major and common confounders (age, gender, body mass index, diabetes, and smoking). Four studies were prospective cohort studies (15-18), which minimized the recall and selection bias. Five studies have more than 15 years of follow-up (13,15-18). Sufficiently long follow-up (e.g. 5-10 years) is necessary because most human cancers have a long subclinical period (38,39).

In summary, our pooled analysis indicates that periodontitis is significantly associated with an increased risk of hematopoietic and lymphatic cancers, especially lymphatic cancers. Moreover, the associations are particularly strong in the population of never smokers and the American population. However, additional prospective studies with a larger inclusion population, improved measurement and classification of PD, longer follow-up duration, and well-designed clinical models adjusted for the major and common confounders (e.g., age, gender, race, smoking status, body mass index, diabetes history) are warranted to confirm present findings. Additional research is expected to assess whether PD prevention and treatment can decrease the incidence of hematopoietic and lymphatic cancers. Findings of this meta-analysis highlight the importance of maintaining good oral health, which may led to a reduced risk of developing hematopoietic and lymphatic cancers.
